# New Horizons in Hydrogels for Methotrexate Delivery

**DOI:** 10.3390/gels7010002

**Published:** 2020-12-30

**Authors:** Ali Dehshahri, Anuj Kumar, Vijay Sagar Madamsetty, Ilona Uzieliene, Shima Tavakol, Fereshteh Azedi, Hojjat Samareh Fekri, Ali Zarrabi, Reza Mohammadinejad, Vijay Kumar Thakur

**Affiliations:** 1 Center for Nanotechnology in Drug Delivery, Shiraz University of Medical Sciences, Shiraz 7146864685, Iran; dehshahria@sums.ac.ir; 2 School of Chemical Engineering, Yeungnam University, 280 Daehak-Ro, Gyeongsan 38541, Korea; anuj.budhera@gmail.com; 3 Department of Biochemistry and Molecular Biology, Mayo Clinic College of Medicine and Science, Jacksonville, FL 32224, USA; sagarchemistry@gmail.com; 4 Department of Regenerative Medicine, State Research Institute Centre for Innovative Medicine, Santariskiu 5, LT-08406 Vilnius, Lithuania; ilona.uzieliene@imcentras.lt; 5 Cellular and Molecular Research Center, Iran University of Medical Sciences, Tehran 1449614525, Iran; shima.tavakol@yahoo.com (S.T.); azeditehrani.f@iums.ac.ir (F.A.); 6 Department of Neuroscience, Faculty of Advanced Technologies in Medicine, Iran University of Medical Sciences, Tehran 1449614535, Iran; 7 Student Research Committee, Kerman University of Medical Sciences, Kerman 7619813159, Iran; hojjatfekri@gmail.com; 8 Pharmaceutics Research Center, Institute of Neuropharmacology, Kerman University of Medical Sciences, Kerman 7616911319, Iran; 9 Sabanci University Nanotechnology Research and Application Center (SUNUM), Tuzla, Istanbul 34956, Turkey; alizarrabi@sabanciuniv.edu; 10 Research Center for Tropical and Infectious Diseases, Kerman University of Medical Sciences, Kerman 7618866749, Iran; 11 Biorefining and Advanced Materials Research Center, Scotland’s Rural College (SRUC), Kings Buildings, West Mains Road, Edinburgh EH9 3JG, UK

**Keywords:** methotrexate, drug delivery, hydrogels, cancer, rheumatoid arthritis, psoriasis

## Abstract

Since its first clinical application, methotrexate (MTX) has been widely used for the treatment of human diseases. Despite great advantages, some properties such as poor absorption, short plasma half-life and unpredictable bioavailability have led researchers to seek novel delivery systems to improve its characteristics for parenteral and oral administration. Recently, great attention has been directed to hydrogels for the preparation of MTX formulations. This review describes the potential of hydrogels for the formulation of MTX to treat cancer, rheumatoid arthritis, psoriasis and central nervous system diseases. We will delineate the state-of-the-art and promising potential of hydrogels for systemic MTX delivery as well as transdermal delivery of the drug-using hydrogel-based formulations.

## 1. Introduction

Methotrexate (MTX) has been considered as a standard therapeutic agent for various diseases including cancer and autoimmune diseases [1]. Regarding the potential effect of folic acid antagonists in the treatment of childhood leukemia, two molecules were synthesized as folic acid competitive inhibitors called aminopterin and amethopterin (methotrexate, MTX). It was found that MTX is less toxic and more stable than its analogue aminopterin. Therefore, it was first used for the treatment of acute leukemia in children [2]. Since the 1940s, the clinical applications of MTX has not been limited to treat various neoplasms such as acute myeloid leukemia, osteosarcomas, non-Hodgkin’s lymphoma, breast and bladder cancers [3,4,5]. The potential of MTX for rheumatoid arthritis was also approved in the mid-1980s. Currently, it is widely used for the treatment of Crohn’s disease [6], multiple sclerosis (MS), myasthenia gravis (MG) [7] and psoriasis [8,9,10]. This drug could also be used for termination of ectopic pregnancy [11]. Interestingly, MTX might be considered as a prodrug since its polyglutamate form could be found in erythrocytes after the clearance of parent molecule from plasma. The effect of such a derivative has been studied for long-term therapy in children with juvenile idiopathic arthritis and juvenile dermatomyositis [12,13]. This wide range of clinical applications of MTX is the result of the various mechanisms by which this molecule affects different pathways and enzymes in the cells. For example, dihydrofolate reductase (DHFR) could be inhibited by MTX due to the structural similarities between folic acid and MTX. It has been shown that DHFR plays a crucial role in the process of thymidylate synthesis. Therefore, its competitive inhibition by MTX results in a decrease in purine and pyrimidine synthesis. This leads to the decrease in cell proliferation particularly in immune cells including T lymphocytes. This mechanism leads to the anti-inflammatory effect of MTX while the inhibition of de novo synthesis of purine and pyrimidine results in the antineoplastic effects of MTX [2]. The other proposed mechanism for MTX is its effect on cyclooxygenase and lipo-oxygenase enzymes which have demonstrated a significant impact on the induction of inflammatory reactions. Some studies are indicating that MTX can selectively inhibit COX-2 in the plasma of patients with rheumatoid arthritis [14,15]. Altogether, the diverse pharmacological effects of MTX could be considered as the result of different mechanisms and pathways affected by the drug.

Another important point which must be considered in the administration of MTX in various diseases is its pharmacokinetic properties. Tremendous investigations have been carried out to reveal the absorption and distribution of MTX as well as its metabolism and excretion. MTX is actively absorbed following the oral administration. However, its absorption profile is capacity-limited. In other words, the saturation of transporter of reduced folates (RFC1) results in the decrease in MTX absorption following the increase in initial doses [16]. Following oral administration of MTX, approximately 10% of the drug is metabolized due to the first-pass effect in the liver. Once MTX reaches the blood circulation, its plasma concentration rapidly reduces. This is the result of RFC1 activity transferring the drug to the cells including erythrocytes, hepatocytes, synoviocytes as well as white blood cells [17]. The major excretion route of MTX is urine while around 30% of the medication is excreted through bile. Following the long-term administration of MTX, its renal clearance decreases due to the increase of adenosine concentration in plasma and consequent effects on the adenosine receptors in the kidney [18]. Following the oral administration, inter-individual variability of MTX bioavailability has been reported [19].

Since MTX is used for various diseases via different routes of administration, the development of novel drug delivery systems to improve its pharmacokinetic properties and targetability is a necessary step for future investigations. Controlling the burst release and the introduction of new routes of administration would be possible if the drug could be formulated using different delivery systems [20,21,22,23,24,25]. Among various drug delivery systems proposed for MTX delivery, great attention has been directed to hydrogels due to their unique and attractive characteristics. These 3D polymeric structures with a great tendency to biological fluids can absorb water leading to swelling. The porous network of hydrogels and the controlling of the swelling process enable these structures to be used as controlled drug delivery platforms. These properties have shown great benefits for MTX delivery. Besides the advantages such as controlling the MTX release profile by hydrogels, this delivery system could be used as an appropriate candidate for transdermal delivery of MTX to treat various diseases including psoriasis. On the other hand, the minimally-invasive and localized drug delivery could be possible using the injectable thermosensitive hydrogels. These delivery platforms can facilitate the formation of syringeable solutions at room temperature as well as gels at the physiological condition. This article aims to review the potential application of hydrogels for MTX delivery. The latest developments in the usage of hydrogel formulations of MTX for the treatment of rheumatoid arthritis, psoriasis, cancer and CNS diseases have been discussed. Additionally, the transdermal delivery of MTX using hydrogel as well as the injectable MTX formulations based on hydrogel platforms has been explained. In the end, the future perspectives for hydrogel-based delivery platforms have been discussed.

## 2. Hydrogels: Types, Properties, and Biomedical Application

Hydrogels are polymeric networks in which their hydrophilic functional groups provide swelling of the system by holding the water in their 3D network while cross-linking of network chains resist to water dissolution [26,27]. The hydrogels are classified based on the source to natural, semi-synthetic and synthetic polymers; however, synthetic polymers can have higher water adsorption capacity, long service life, and gel strength based on their monomers. The synthetic hydrogel more efficiently than natural ones can be tailored and functionalized for the special target.

Noteworthily, they can be classified based on their composition to homopolymeric, copolymeric, semi-interpenetrating polymer network (IPN), and multipolymer IPN hydrogels. In brief, homopolymeric and copolymeric are derived from a single and two or more diverse monomer species with at least one part of the hydrophilic component. Semi IPN and multipolymer IPN are composed of two independent cross-linked polymers; however, in semi IPN, one component can be non-cross-linked and just have one cross-linked polymer [28]. Besides composition, a hydrogel can be categorized based on its configuration to amorphous, semi-crystalline, and crystalline [29]. Based on their applications in medicine, they can be categorized into implantable, injectable, sprayable and endogenously, exogenously and bio-responsible triggered drug release hydrogels [30,31,32].

As mentioned earlier, the cross-linking of hydrogels is critical to avoid water dissolution. There are two types of chemical and physical cross-linking agents; physical cross-linkers provide physical interactions and polymer chain entanglements [33]. By altering the degree of cross-linking, the mechanical strength is modulated. However, they may have different network electrical charge such as non-ionic (neutral), ionic, amphoteric electrolyte (containing both acidic and basic groups), zwitterionic (containing both cationic and anionic groups).

Several methods for the preparation of hydrogels include solution polymerization/cross-linking, bulk polymerization, suspension polymerization or inverse-suspension polymerization, grafting to support, and polymerization by irradiation (microwave and ultraviolet techniques), complex coacervation, hydrogen-bonding, enzymatic, self-assembly, etc. In other words, polymeric hydrogels are prepared by polymerization of hydrophilic components and or functionalization of existing polymers [26]. The hydrogel can respond to physical and chemical environmental conditions. In other words, temperature, electric and magnetic fields, light, pressure, and sounds are the physical stimuli responses [34,35,36]. At the same time, pH, ionic strength, solvent composition, and molecular species are chemical stimuli responses that change the swelling or de-swelling extent of the hydrogel in the form of volume collapse or phase transition [37,38,39,40]. These responses provide a variety of applications.

Hydrogels can be used in diverse industries; for example, agriculture, artificial snow, coal dewatering, sealing, contact lenses, hygiene products, pharmaceuticals, drug delivery systems, regenerative medicine [41,42], biomedical applications, wound dressing, separation of cells and biomolecules, diagnostics, and biosensors [43,44,45,46,47].

Hydrogels in biomedical applications should be biocompatible. In other words, they must be bio-safe and bio-functional. In the content of bio-functionality, it might be said that the hydrogel should have the ability to function for the specific designed mission. This context usually is important for tissue engineering and drug delivery applications [48]. There are many patents in the field of application of hydrogels in biomedicine, but just a few of them have reached the market.

In 1971, the FDA approval for contact lenses of poly-2-hydroxyethyl methacrylate (PHEMA) was issued. The method preparations for the soft contact lenses (hydrogel-based lenses) are spin-casting, mold-casting, lathe-cutting, etc. However, the silicon-based hydrogel contact lenses are more favorable in the market due to more user-friendly and higher oxygen permeability but suffer from protein deposition [49]. Besides vision correction, they can act as a drug reservoir for eye diseases. For example, by incorporating drug- colloidal structures, ligand, and multilayer including hydrogels in the contact lenses can deliver H1-antihistamines in a time-dependent manner. Furthermore, hydrogels may be applied for wound dressing application. An ideal wound dressing should adsorb the toxin and exudates, preventing infection and extra heat while providing gas penetration and wettability. In 1994, the transparent thin film of wound dressing was patented by Cartmell and Sturtevant. It was made from polypropylene glycol or polyethene glycol, and isophorone diisocyanate [50]. However, later, others added other material such as the antibiotic and wound healing agents.

Moreover, hydrogels owing to their porous structure, can load drugs and sustain release them into the environment. The mechanisms behind the release of drugs by hydrogels are environmentally-responsive release and chemically, diffusion, and swelling controlled release [51]. One of the successful products is vaginal insert Cervidil. It is made from polyethene oxide/urethane polymer and is containing 10 mg dinoprostone in 1995 [52]. The following section explains the MTX delivery by the hydrogel-based structures.

## 3. Hydrogels for Methotrexate Delivery

Hydrogels of various compositions of synthetic and natural components have been widely investigated for MTX delivery systems [53,54,55,56,57]. Major properties addressed to hydrogels for MTX delivery are thermo-stability and sustained release of a drug in vivo. Both features can be modified and are controlled according to chemical crosslinking, the addition of nanostructures, for instance, carbon nanotubes, or applying various additional stimuli, such as temperature, electric potential, ionic strength, and pH [56,58].

Thermo-stability is achieved when a solution of a hydrogel changes its state from the liquid solution to gel after injecting it in vivo upon the stimulation of a body temperature. Thermo-stable hydrogels have been tested in different diseases in vivo, for instance, osteosarcoma, rheumatoid arthritis, and ocular inflammatory diseases [54,59,60,61,62]. Controlled release of MTX depends on the biodegradability of a hydrogel in vivo. Chitosan is mostly favourable natural polymer for thermosensitive hydrogel formation, as it possesses antibacterial, biocompatible and biodegradable properties [56,63]. One of the examples of the commercially available chitosan hydrogels (ChitoClear^®^) was used for hydrogel-nanoparticle system preparation with MTX by ionotropic gelation technique. These hydrogels have been tested for stability during electron bombardment in transmission electron microscopy, which resulted in perfect stability of a hydrogel system with specific drug release kinetic [64]. Modification of chitosan hydrogels with black phosphorus nanosheets (BPNs) also provided promising results in a case of rheumatoid arthritis. These systems combined BPNs with platelet-rich plasma (PRP)-chitosan thermoresponsive hydrogel, where BPNs could generate local heat in response to infrared irradiation and provide raw materials for osteanagenesis. PRP effectively improved adhesion and capacity of mesenchymal stem cells to chitosan hydrogels, which can serve as additional protection for articular cartilage [59].

Chitosan and hyaluronic acid (HA) have been extensively studied in hydrogel systems due to their biodegradability and biocompatibility. HA conjugated with MTX (HA-MTX) systems have been tested in vivo arthritis models, psoriasis [65,66,67]. Therefore, hydrogels developed for sustained release of MTX are promising in different disease areas and provide more advantageous properties as compared to direct injection of MTX to diseased tissues, or oral consumption.

### 3.1. Transdermal Methotrexate Delivery

There are various strategies to deliver therapeutics for human diseases therapy. Oral, rectal, ocular, vaginal, and transdermal routes are considered to design the drug delivery systems. Hydrogels possessing unique properties widely used for transdermal drug delivery [68,69,70]. Recently, microneedles are considered as an enhanced delivery technique and applying this method for transdermal drug delivery provides a promising alternative to previous methods [71]. It is well established that balance between drug loading and mechanical strength of the microneedle is important. Hydrogel-forming microneedles making by biocompatible, non-ionic triblock amphiphilic thermosensitive copolymer, could pass these barriers. Drug delivery from these microneedles can be controlled by altering the crosslink density of the polymer [72].

**Figure 1:**
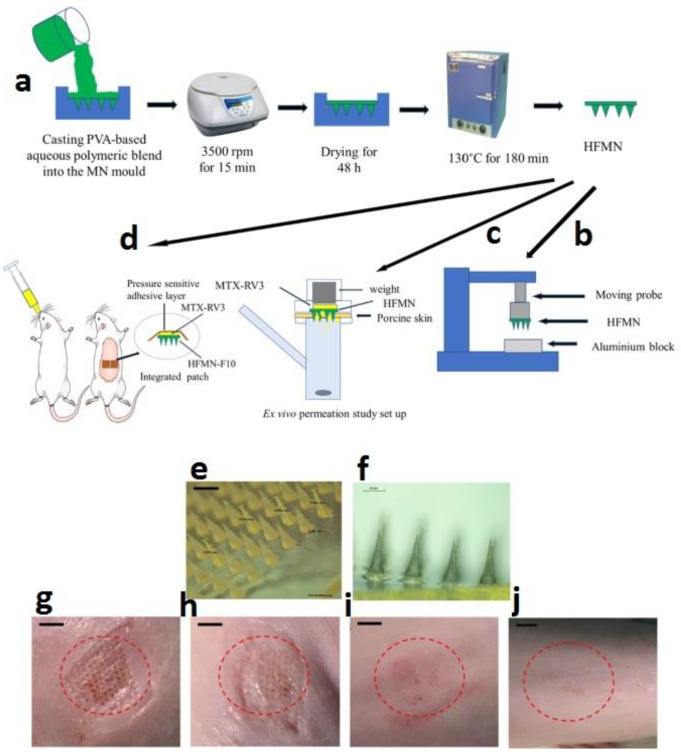
(**a**) Schematic representation of preparation procedures for hydrogel-forming microneedle arrays (HFMN). PVA-based polymeric blends (green) and MTX (yellow). (**b**) Experimental setup for measuring the mechanical strength of HFMN. (**c**) Schematic diagram for the experimental setup for the ex vivo permeation studies. (**d**) The experimental setup for the in vivo study, where two integrated patches were applied to the back of the rat and MTX aqueous solution administered orally. (**e**) Microscopic images of HFMN-F10 before insertion into skin, the black scale bar represents a length of 0.5 mm. (**f**) Swollen intact HFMN-F10 after removing from rats skin following 24 h application. (**g**) Digital images showing the micro holes created by HFMN in rats skin immediately after HFMN removal and that no irritation at the application site but only mild erythema. (**h**) The microholes visibly closed after 30 min from removing the HFMN. (**i**) The microholes cannot be identified visibly after 2 h from removing the HFMN. (**j**) No sign of infection (redness, swelling, pus, oozing or weeping) at the application site even after 24 from removing the HFMN. [74]. Reprinted with the permission from International Journal of Pharmaceutics. Copyright Elsevier, 2020.

Transdermal delivery presents an attractive alternative administration route [73]. However, MTX passive permeation through the skin is hindered by the skin barrier and MTX physicochemical properties. In the recent study by Tekko et al. [74], they developed a novel hydrogel-forming microneedle arrays (HFMN) and a patch-like reservoir loaded with MTX (MTX-RV). Both the HFMN and MTX-RV were completely characterized and then combined to form an integrated patch. Their results showed the MTX-RV incorporated a high dose of MTX (150.3 ± 5.3 µg/mg) without any precipitation. Eventually, they could develop a promising minimally invasive transdermal drug delivery system that could overcome the skin barrier and deliver MTX in a sustained manner (Figure 1).

Additionally, Sivaraman et al. [75] developed a novel in situ forming hydrogel microneedles using a biocompatible non-ionic triblock amphiphilic thermosensitive copolymer. MTX delivery using the prepared microneedles was evaluated in the porcine ear and dermatomed human skin. The results showed the sol-gel transition in the porated site of the skin at 32 °C and the in situ formed hydrogel microneedles embedded within the microporated skin site. The formulation provided a steady and sustained delivery of MTX. Likewise, Alvarez-Figueroa et al. [76] studied MTX transdermal administration for treatment of psoriasis by iontophoretic delivery from two types of hydrogel and passive delivery from two types of the microemulsion. For passive delivery assays, they used both water/oil and oil/water microemulsions. The effectiveness of the delivery system was higher from oil/water systems. At the end of all assays, they could find that significant amounts of MTX were detected in the skin. Therefore, they concluded that both hydrogels and microemulsions may be of value for the topical administration of MTX in the treatment of psoriasis.

### 3.2. Injectable Hydrogels for Methotrexate Delivery

Low molecular weight injectable hydrogels, due to their inherent biocompatibility and physical properties, are considered advantageous over pre-formed implants because of their minimal invasiveness. These injectable hydrogels can be delivered into the body through a catheter or by direct injection via syringe [77,78]. Injectable thermosensitive hydrogels undergo a sol-gel phase transition in response to temperature change [79] and facilitate syringeable solution at room temperature and gelling at physiological temperature followed by minimal invasion and localized release of drug at the desired site of action. The enhanced gelation under a biological environment and thereby improved overall biological performance of a hydrogel is a significant factor for this purpose. For this, both natural and synthetic polymers have been used to control the delivery of drugs in various biomedical applications [80,81]. However, the burst release of the drug prevents the clinical application of such type of hydrogels. Therefore, Dang et al. prepared CS-based microspheres (CMs) and developed a double-component injectable system by incorporating CMs into CS-α,β-glycerophosphate hydrogel (CS-HG) to eliminate this limitation [82]. CMs-CS-HG exhibited excellent injectability and drug-loaded CMs led to the localized release of the drug. Further, both in vitro and in vivo MTX release analyses showed long-term sustained release of MTX from MTX-loaded CMCs-CS-HG. Here, double-component CMs-CS-HG sol was easily be injected into dorsal subcutaneous tissue. For comparative analysis, three separate injections of NaCl solution, CMs suspension, and CMs-CS-HG sol were used. After injection, CMs-CS-HG sol was observed to show quick gel formation (i.e., oval-shaped protrusions) at physiological temperature environment. Further, HE staining also confirmed this semi-solid gel formation at the site of injection, whereas diffusion of CMs throughout the subcutaneous tissue. However, after injection, no macroscopic inflammation was observed in all three groups around injected-site. Moreover, the obtained double-component CMs-CS-HG maintained excellent injectability, biocompatibility, and prolonged drug release time as compared to the single component CMs or CS-HG.

**Figure 2:**
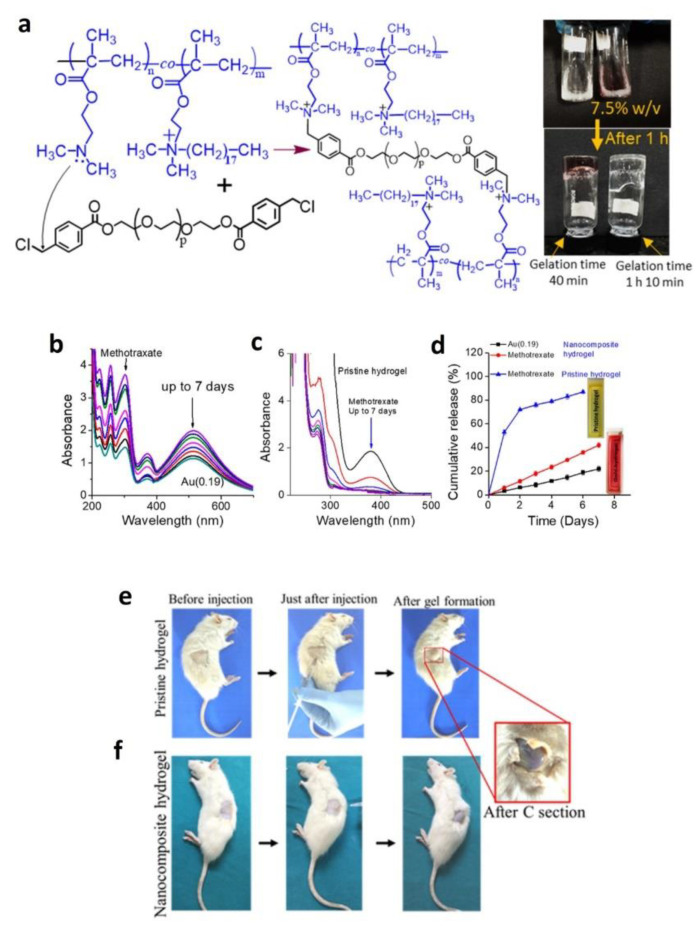
(**a**) Schematic illustration and digital photographs of the hydrogel formation (prepolymer solution = 7.5% *w*/*v*). The vials were inverted after a certain time for recording the photographs. (**b**,**c**) Lowering of UV-Vis spectra intensity of entrapped methotrexate (MTX) and AuNPs (λmax = 514 nm) with incubation period. (**d**) Cumulative release (%) of MTX and AuNPs with incubation period (at pH 7.4). (**e**,**f**) Injection of a prepolymer containing no NPs and Au(0.19) NPs [83]. Reprinted with the permission from Biomacromolecules. Copyright American Chemical Society, 2020.

In another study, Nutan et al. reported on the formulation where gold nanoparticles (AuNPs) accelerated rapid formation of injectable nanocomposite hydrogels in vitro and in vivo with enhanced modulus, cell attachment, cell proliferation, and cytocompatibility compared to pristine hydrogel system. These Au (0.19) NPs-based nanocomposite hydrogels showed sustained co-release of MTX (anti-rheumatic arthritis drug) and AuNPs [83] over an extended time duration (see Figure 2b,d), whereas a burst-release leading to a sustained release of MTX was observed from pristine hydrogel (see Figure 2c,d). Further, MTX drug release was faster than that of AuNPs and this was due to the relatively slow diffusion of hydrated NPs as compared to a low-molecular-weight drug (see Figure 2d). Moreover, this slow and sustained release ability is attributed to low swelling behavior, high crosslinking density, and low degradation rate of the nanocomposite hydrogel.

It is known that 5-Fluorouracil (5-FU) as a model cytotoxic drug (i.e., anticancer drug) exhibits poor selectivity to the cancer cell and its systematic administration exposes normal cells to drug-induced toxicity through oral or injection process. Therefore, Mohammed et al. developed CS-based thermosensitive hydrogels loaded with 5-FU and crosslinked with various agents such as β-glycerophosphate (β-GP), pluronic F127, and hydroxyapatite. In addition, MTX was added to 5-FU to obtain the synergistic effect of both drugs. CS-based hydrogel crosslinked with β-glycerophosphate and 10% pluronic F127 in combination exhibited the most suitable physicochemical properties and release behavior [63]. Carbon nanomaterials have also been applied to various drug delivery systems and cancer/tumor therapies. In this way, carbon nanotubes (CNTs) were incorporated into a thermosensitive and injectable hydrogel composed of CS and β-GP. The developed hydrogel loaded with MTX (CS-β-GP-CNT-MTX) was a syringeable solution at room temperature and became a solidified gel at physiological temperature (i.e., body temperature). Hydrogels having 0.1% CNT were not toxic to 3T3 cells and demonstrated a reduced release rate of MTX as compared to control hydrogel (without CNT). Furthermore, the CS-β-GP-CNT (0.1%)-MTX hydrogel system improved the MTX antitumor activity [56]. Graphene NPs (GNPs) having unique optical, electrical, and mechanical properties demonstrated good potential in drug delivery applications. MTX loaded CS/GNPs-based thermo-sensitive injectable hydrogels could easily deliver anticancer activity of MTX as a locally targeted and sustained chemotherapy through a simple injection. It exhibited a slow and more controllable release of MTX compared to control. MTX loaded CS/GNPs hydrogels could inhibit the growth of breast cancer cells [84].

Intra-articular delivery of drugs at the joints directly is a promising approach to reduce undesirable side effects that are associated with systematic delivery of drugs. Miao et al. prepared an injectable thermosensitive poly (ε-caprolactone)-poly (ethylene glycol)-poly(ε-caprolactone) (PCL-PEG-PCL) (PEP) hydrogels for controlled delivery of MTX for intra-articular drug delivery for treating arthritis disease. The synthesized PEP copolymers aqueous solutions showed rapid in-situ gel after the injection and exhibited good biocompatibility. The results showed controlled release of loaded MTX from PEP network and following to intra-articular injection, PEP hydrogels reduced the clearance rate of MTX in the joint cavity [85]. In another study, MTX-loaded nanostructured lipid carrier (NLC)-based injectable smart gel for effective rheumatic disease treatment. The optimized NLC-based gel (F-10) was found thermo-sensitive and showed a 92.41% drug release at 108 h. Further, MTX was homogeneously distributed in this optimized gel and showed injectability through 18 gauze-syringe needles. Moreover, MTX-NLC based smart gel exhibited a significant reduction in rat joint swelling during 28 days [86]. In a recent study, MTX was formulated with hydroxypropyl methylcellulose K4M (HK4M), polycarbophil (PCL), and pluronic F-127 and this developed in-situ thermoresponsive and injectable gel (M4) exhibited 93.26 ± 2.39% release of MTX at 96 h. Additionally, the gel was biocompatible at the injection site and in vivo analyses on Wistar rats showed a considerable decrease in paw oedema during 28 days [87].

## 4. Methotrexate-Loaded Hydrogels for Arthritis Therapy

MTX is a first-line disease-modifying drug proposed for treating the inflammatory forms of arthritis [88]. Rheumatoid arthritis (RA) and osteoarthritis (OA) are one of the leading forms of arthritic diseases, causing global disability [89], where RA is a chronic autoimmune disease and OA has been considered as a load-bearing disease, with low-grade inflammation [90,91,92]. MTX is the initial preferred drug for rheumatic diseases and is considered to be the gold standard for treatment of RA, used for more than three decades [93], however, it has been also proposed for treating OA [94]. MTX efficiently reduces pain, swelling and slows down the arthritic disease progression [90].

Oral administration hampers MTX efficacy by poor solubility, short plasma half-life and might cause gastrointestinal toxicity, which is therefore being changed to parenteral administration [88,95]. MTX-carrying hydrogel systems are more potential than the direct injection, as MTX release can be controlled, enabling its prolonged effects. For instance, thermo-sensitive hydrogels, composed of disulfide-crosslinked polyethyleneimine (PEI-SS) nanoparticles loaded with MTX and indomethacin were injected into rat collagen-induced RA knees, demonstrated significant reduction of joint swelling, bone resorption and inflammatory cytokine expression after the controlled release of both drugs, as compared to single MTX/indomethacin injections [96]. Additionally, same hydrogels were additionally modified by incorporating small interfering RNAs (siRNA), targeting matrix metalloproteinase 9 (MMP-9), which is involved in extracellular matrix degradation [97]. After intra-articular injection into arthritic mice, hydrogels effectively reduced joint swelling, expression of MMP-9 and even restored morphological parameters of joint close to normal [98]. Furthermore, HA–MTX conjugates are popular due to specific HA binding to CD44 in macrophages, which prevents severe inflammation in RA mice and are considered promising therapeutic agents for RA [99]. As an example, high molecular weight product DK226, consisting of both HA and MTX has shown promising, anti-arthritic effects in rat RA model, by reducing the swelling and synovial inflammation was proposed not only for treating RA but also OA [66]. Another hydrogel system proposed for MTX delivery contained click-crosslinked HA (Cx-HA) depots, which were cross-linked between tetrazine-modified HA and trans-cyclooctene-modified HA. These hydrogels demonstrated efficient delivery of MTX into rat RA joints. They persist at the joint site, as compared to MTX alone or HA-MTX, maintain therapeutic MTX concentrations for an extended period and significantly restored RA-affected joints [65].

**Figure 3:**
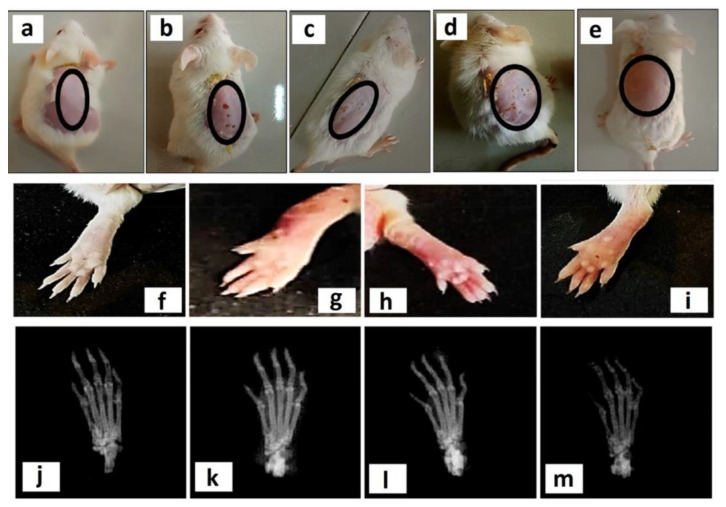
(**a**–**e**) Visual representation of skin irritation is shown in the second row of four different treatments, that is, (**a**) normal control (no treatment), (**b**) 0.8% formalin treated as a negative control, (**c**) free MTX-loaded hydrogel, (**d**) free MTX-loaded hydrogel with EO, and (**e**) MTX-NMs along with EO-based hydrogel. (**f**–**i**) Photographs of representative mice hand paws among the four treatment groups (plantar view). (**j**–**m**) X-ray images of hind paws among the different treatment groups. (**j**) normal control (not immunized with CFA and treated with normal saline), (**k**) negative control (immunized with CFA and treated with normal saline), (**l**) rheumatoid arthritis (RA) mice model treated with free MTX-based hydrogel, and (m) RA mice model treated with MTX-NMs + EO-based hydrogel [57]. Reprinted with the permission from ACS Nano. Copyright American Chemical Society, 2020.

In addition to intra-articular injection, MTX-carrying hydrogel systems are also being applied trans-dermally. Different lipid-based systems have been suggested as MTX carriers, however, they exhibited poor stability [100,101], therefore, additional carriers have been developed. For instance, MTX-loaded aspasomes exhibit high drug entrapment efficiency with controlled release and adequate drug permeation in the trans-dermal application. The developed aspasomes were prepared using carbopol, loaded with MTX and used for arthritic rats, has revealed reduced paw diameter, cartilage damage, inflammation, as compared to arthritic control rats [55]. Moreover, nano micelles are also potent carriers for MTX. Polycaprolactone-polyethylene glycol-polycaprolactone (PCL-PEG-PCL)-based self-assemble nanomicelle carrier system, prepared by nanoprecipitation technique for MTX delivery has shown promising results in RA mice model. It was shown that after loading nano micelles into the carbopol-934 hydrogel, together with eucalyptus oil for better penetration, resulted in higher accumulation of nano micelles in the inflamed tissues, as well as reduction of inflammatory cytokine expression (Figure 3) [57]. Transdermal approaches are attractive as non-invasive treatment with reduced side effects and MTX-related toxicology. Therefore, hydrogel systems for MTX delivery have become one of the most potential spheres for local drug delivery in RA joints, without affecting other tissues.

## 5. Methotrexate-Loaded Hydrogels for Psoriasis Therapy

**Figure 4:**
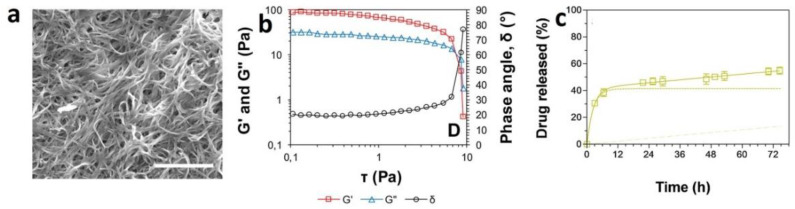
(**a**) SEM images of xerogels of 1·methotrexate. Scale bar represents 10 μm. (**b**) Elastic modulus (G′), loss modulus (G″) and phase angle (δ) as a function of shear stress. (**c**) In vitro drug released (%) along time from gels. Drug release in all cases followed a Two-Phase Exponential Association model. The first phase release is shown in dotted line, the second phase release is shown in dashed line, and the cumulative amount of drug released from both phases is shown in continuous line. [115]. Reprinted with the permission from Colloids and Surfaces B: Biointerfaces. Copyright Elsevier, 2019.

The first investigation on the application of folic acid antagonist for the treatment of psoriasis was reported in 1958, where the low doses of aminopterin led to a significant improvement in the patients with psoriasis [102]. However, the wide clinical application of MTX for its anti-inflammatory effects was reported in the 1980s [12]. Psoriasis could be defined as an inflammatory autoimmune disease which affects skin resulting in the formation of lesions in several parts of the body including elbows, knees, scalp and back. Various types of cells and immune mediators are involved in the progression of the disease. For example, autoantigens such as LL-37 peptide are secreted from keratinocytes leading to the activation of T cell lymphocytes and dendritic cells. This activation results in the secretion of various cytokines including IL-17, IL-20, IL-22, IL-23 and IFN-γ which promotes the proliferation of keratinocytes [103,104,105]. To control the diverse immune cascades associated with the progression of the disease, the different drug has been suggested including immunosuppressant and corticosteroids. These medications could be used as systemic or topical dosages forms. However, the systemic administration of such medications is limited to the severe cases not responding to topical treatments [106,107]. In addition to the above-mentioned drugs, oral or parental forms of MTX have also been administrated for severe cases of plaque psoriasis. The application of MTX for severe psoriasis not responding to other treatments has been approved by FDA and EMA [108]. Although great achievements have been observed following the administration of MTX in patients with psoriasis, there are some concerns regarding its long-term application in terms of drug resistance. In addition, variable adsorption and fluctuations in bioavailability as well as non-selective toxic activity in high doses have led researchers to seek topical dosage forms for psoriasis treatment [109]. Various types of formulations have been developed for topical administration of MTX including liposomes, nanogels [110,111], nanostructured lipid carriers as well as hydrogels [112,113]. For example, Ali and colleagues prepared a liposomal MTX hydrogel from DPPC, soy PC, egg yolk PC, and cholesterol and tested the formulation either topically or as targeted delivery [112]. The gel formulation was used every day followed by the irradiation from a 650 nm diode laser for 3 months in albino mice. The results revealed that this formulation of MTX hydrogel was useful for psoriasis treatment and did not lead to systemic toxicity. In another study conducted by Katare group [109], microemulsion based MTX hydrogel was prepared and evaluated in ex vivo and in vivo models. The results indicated that the formulation is able to deliver MTX at the desired layers of the skin with reduced systemic toxicity [109]. In another investigation, the effectiveness and adverse reactions of a topical MTX (0.25%) preparation in a hydrogel base were evaluated in the patients suffering from palmoplantar psoriasis [114]. Although the formulation was well tolerated, the effectiveness in controlling the lesions was not significant. Therefore, higher drug concentrations as well as modification of formulation were suggested for better clinical results [114]. Recently, nanostructured supramolecular hydrogels using dicationic imidazolium-based amphiphile was prepared as topical formulation for psoriasis treatment [115]. The results of ex vivo study demonstrated successful skin permeation of the drug and its retention inside the target site. In vivo experiments indicated that this formulation decreases the hyperplasia and tissue damage more that the formulations such as solutions (Figure 4) [115]. Altogether, hydrogels could be considered as suitable candidates for topical delivery of MTX.

## 6. Methotrexate-Loaded Hydrogels for Cancer Therapy

Cancer is the second leading cause of death after cardiovascular worldwide, which accounts for millions of deaths every year [116] Although significant progress has been achieved in the cancer treatment field, numerous issues must be addressed to improve cancer therapy [117]. Scientists across the globe are working hard day and night in developing efficient therapies to overcome the problems concerning the existing conventional therapies [118]. Numerous technologies are currently undertaking clinical trials, and some have already been introduced into practice [119].

Nanomedicine contributes a great impact on the development of biocompatible nanomaterials for diagnostic and therapeutic purposes [120,121,122]. Among several platforms, hydrogels possess their role in the drug delivery field in several diseases, including cancer [78,123,124]. Due to recent advances in hydrogel-based drug delivery systems, this field is getting more and more attractive to exploring further [125]. Hydrogels provide a vast range of benefits in drug delivery applications, such as easy preparation, improved high local drug concentration at the tumor site, prolonged drug retention time, reduced drug dose in vivo, good biocompatibility, and improved patient compliance [126,127].

**Figure 5:**
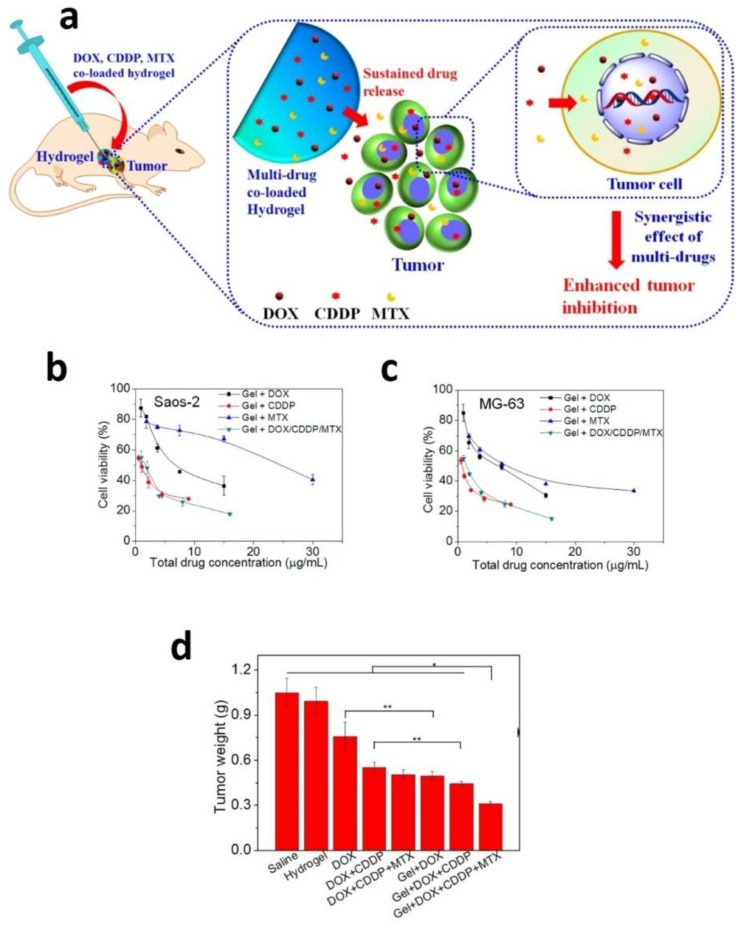
(**a**) Schematic illustration of the localized and sustained co-delivery of DOX, CDDP, and MTX using PLGA-PEG-PLGA hydrogels for synergistic treatment of tumor. (**b**,**c**) Cytotoxicity of the hydrogels containing the single drug or multiple drugs against Saos-2 or MG-63 cells, after incubation for 48 h. (**d**) Tumor weights obtained from the BALB/c nude mice bearing human osteosarcoma Saos-2 xenografts after single injection to the vicinity of the tumors at day 16. Data were presented as mean ± standard deviation (*n* = 6). (* *p* < 0.05, ** *p* < 0.01) [133]. Reprinted with the permission from ACS Applied Materials & Interfaces. Copyright American Chemical Society, 2015.

MTX is an anticancer and immunosuppressive drug used to treat breast, blood, bone, lung, head, neck cancer, osteosarcoma, and treat rheumatoid arthritis psoriasis [60,128,129]. However, its poor water solubility and side effects limit its usage in the clinic. Hence, several scientists developed MTX loaded hydrogel to improve solubility and lower the side effects and treat cancer [61,130]. For example, a novel MTX and alendronate co-loaded mPEG45–PLV19 containing thermosensitive hydrogel was developed by scientists, demonstrating synergistic inhibition osteosarcoma with sustained release of drugs [131]. In another study, investigators developed in situ MTX loaded gelatin and poly(vinyl) alcohol (Gel/PVA) hydrogel and used to treat colorectal disorders [132]. Similarly, Ma et al. developed a new approach of multiple drugs (doxorubicin, cisplatin, and MTX) co loaded thermosensitive PLGA-PEG-PLGA hydrogels for the localized treatment of osteosarcoma (Figure 5). Subsequently, a single dose injection of these multiple drug-loaded hydrogels into Saos-2 bearing human osteosarcoma displayed superior tumor growth inhibition. Importantly these multiples drug-loaded hydrogels did not show any toxicity in the mice body change. The histopathological examination of the major organs directed that the localized treatments showed no apparent damage to the normal organs and less systemic toxicity [133]. Further, scientists prepared MTX loaded hydrogel using PAAm-g-Gg (gum ghatti) polymer, which was successfully synthesized by free radical polymerization. This MTX loaded hydrogel protects from premature release and allows the MTX release at the desired site. Therefore, these gum ghatti hydrogel NPS are the prospective system for colon delivery of MTX for colon cancer chemotherapy [134].

Recently, electric-field sensitive hydrogels are of great interest for several investigators from the features of their usage in numerous biomaterials applications. These hydrogels were able to control drug release under voltages, which offers enormous benefits for the drug delivery systems [135]. Similarly, MTX, rhodamine B co-loaded and near-infrared stimulated hybrid hydrogel patches were developed using alginate (Alg), polyacrylamide (PAAm), for thermoresponsive MTX delivery [136]. Scientists developed a sensitive, rapid method for measuring MTX in biologic fluids using hydrogels based solid-phase radioimmunoassay. From this method, the authors can measure drug concentrations of less than 1 ng/mL [137].

Hybrid hydrogels from the magnesium oxide and natural polymer-based copolymer of acrylic acid (AAc) and xanthan gum (Xan) were prepared using radiation-induced copolymerization cross-linking procedures and used as a drug delivery system. Integration of MgO into (Xan-AAc) hydrogel improved the drug loading efficiency and enhanced the (MTX) release to reach the maximum in the simulated intestine with a sustained drug release profile [138]. Both psyllium and MTX possess anticancer natures, and psyllium can be appropriately tailored to prepare the hydrogels. So, researchers used psyllium for developing the hydrogels for delivery of MTX in a sustained and controlled manner [139].

A novel hydrogel was prepared for the local delivery of multiple antineoplastic agents (MTX, doxorubicin, and mitoxantrone), demonstrating the different release types. Here the authors chemically modified alginate into low molecular weight oligomers and cross-linked with a biodegradable adipic dihydrazide spacer, which ultimately forms biodegradable hydrogels. MTX, doxorubicin, and mitoxantrone (a three-model drug system) were loaded into the hydrogel through three mechanisms. MTX was integrated within the hydrogel pores, which was released by diffusion. Doxorubicin was chemically added to the polymer backbone using a hydrolytically labile linker, which was released by chemical hydrolysis. Finally, mitoxantrone was ionically complexed to polymer, was released with disconnection of the complex. Hence, these three release mechanisms could potentially deliver a wide range of drugs based on their chemical structure [140]. Another study, researchers developed and characterized MTX loaded de-esterified tragacanth-chitosan hydrogels as a novel carrier to improve drug efficacy and targetability [141]. Similarly, MTX-loaded pH-responsive magnetic hydrogel beads based on Fe_3_O_4_ nanoparticles and chitosan were prepared through a very facile, economical and environmentally friendly one-step gelation process. MTX-encapsulated magnetic chitosan hydrogel beads showed good cytocompatibility and high anti-tumor activity [142]. In summary, MTX-loaded hydrogels showed their potentials for the treatment of cancer.

## 7. Methotrexate-Loaded Hydrogels for Central Nervous System Diseases Therapy

The mechanistic roles on MTX-loaded chitosan-based hydrogel nanoparticles intended for central nervous system (CNS) drug delivery were considered in studies. Previous studies showed that Chitosan-based hydrogel nanoparticles could provide a higher concentration of MTX in the brain. Jahromi et al. [143] demonstrated that following administration of MTX containing chitosan nanogel intravenously, spherical nanogels (mean diameter of <200 nm), zeta potential (22.8 ± 6.55 mv), Loading efficiency (72.03 ± 0.85), and loading capacity (1.41 ± 0.02) produce a considerably higher brain concentration compared with the simple solution. They give one group a verapamil dose 30 min before MTX. They could show a higher brain concentration of MTX in this group. Moreover, they could display that less than one hour after drug administration, nanogels can help MTX passage like “Trojan horse effect”. It can provide a high concentration of drug in contact with the blood–brain barrier (BBB). It has to be noticed that during the extended time, this nanogel could cross the BBB and release a substance beyond that.

Drug delivery to the NS has always been a big challenge, particularly for MTX because of the poor BBB passage. Recent studies have been done on intranasal drug administration for brain drug delivery intentions. This is because this method of drug administration is noninvasive, being independent of blood and the gastrointestinal tract. By this method of administration, therapeutic agents can bypass the BBB and hepatic first-pass effect, which ultimately leads to a low dose of the drug and fewer side effects.

Recently, applying MTX-loaded hydrogel nanoparticles via intranasal delivery was studied by means of survey. Jahromi et al. [144] showed that for the treatment of primary CNS lymphoma, MTX-loaded hydrogel nanoparticles produced a significantly higher concentration of MTX in the brain but not in the plasma when compared to the free drug solution. Drug targeting efficiency and direct transport percentage for nanogel (as a test) and free drug solution (as control) were 424.88% and 76.46% and 34,842.15% and 99.71%, respectively. In comparison to intravenous administration of the same nanogel, it was indicated that intranasal administration significantly increases the brain concentration of MTX.

## 8. Conclusions and Future Perspectives

MTX has been widely used for the treatment of cancer as well as inflammatory and CNS diseases. Various drug delivery platforms have been proposed for MTX including hydrogel-based formulations. This delivery system provides a substantial opportunity to overcome some major drawbacks of MTX administration such as its low bioavailability, dose-dependent adverse reactions and resistance following the long-term applications. The specific properties of hydrogels enable this system to release MTX in a controlled manner. In addition, the injectable hydrogel is promising formulations for the preparation of parenteral controlled delivery of MTX. Additionally, the hydrogel formulation of MTX could be considered as a brilliant approach for transdermal delivery to reduce the side effects associated with the systemic administration. On the other hand, the hydrogel formulation of MTX opens up new horizons for novel routes of administrations.

However, several major points must be considered for further developments of hydrogels. The drug release rate, cross-linking degree and loading efficiency could be controlled through different cross-linking chemistries as well as preparation methods. In other words, the improvements of MTX novel formulations based on hydrogel platforms need more precise adjustments in their preparation procedures. Non-immunogenic and biocompatible materials for the preparation of hydrogel-based formulations are a critical prerequisite for their further clinical translation. In another words, only the formulations prepared by biocompatible starting materials via simple and well-established chemistry have the chance for clinical applications. Additionally, batch-to-batch reproducibility and scalable production method guarantee their large-scale production in a well-controlled and high throughput manner. Another critical issue in the hydrogel formulations of MTX is the delivery of an active compound to the precise site of action. This highlights the functionalization of hydrogels with targeting ligands. MTX has shown pharmacological activity in different cells through various mechanisms. Therefore, it is necessary to direct the drug to the desired site of action. Therefore, fabricating targeted hydrogels via simple and efficient process may reduce some side effects associated with the impact of the drug on healthy tissues.

Co-delivery of small molecule drugs such as MTX and oligonucleotides might be considered as a novel approach to overcome some adverse reactions associated with the long-term application of MTX. Oligonucleotide-based therapeutics can decrease the drug resistance particularly in long-term usage of the drug. Therefore, several oligonucleotides including siRNA, plasmid encoding shRNA and miRNA could be used in combination with MTX [98,145,146,147,148]. The properties of hydrogels must be modified to make them as suitable oligonucleotide carriers. In other words, these systems must be engineered in a smart way to be able to carry a conventional drug (e.g., MTX) and oligonucleotide therapeutics. This combination increases the pharmacological impact of the drug without increasing its initial dose. Hence, such novel cocktails of drug and gene may reduce the adverse reactions facilitating their clinical applications even for new purposes.
